# Rationale and design of the precise percutaneous coronary intervention plan (P3) study: Prospective evaluation of a virtual computed tomography‐based percutaneous intervention planner

**DOI:** 10.1002/clc.23551

**Published:** 2021-03-03

**Authors:** Sakura Nagumo, Carlos Collet, Bjarne L Norgaard, Hiromasa Otake, Brian Ko, Bon‐kwon Koo, Jonathon Leipsic, Daniele Andreini, Ward Heggermont, Jesper M Jensen, Yu Takahashi, Abdul Ihdayhid, Zinlong Zhang, Emanuele Barbato, Michael Maeng, Takuya Mizukami, Jozef Bartunek, Adam Updegrove, Martin Penicka, Campbell Rogers, Charles Taylor, Bernard De Bruyne, Jeroen Sonck

**Affiliations:** ^1^ Cardiovascular Center Aalst OLV Clinic Aalst Belgium; ^2^ Department of Cardiology Showa University Fujigaoka Hospital Yokohama Kanagawa Japan; ^3^ Department of Cardiology Aarhus University Hospital Aarhus Denmark; ^4^ Division of Cardiovascular Medicine, Department of Internal Medicine Kobe University Graduate School of Medicine Kobe Japan; ^5^ Monash Cardiovascular Research Centre Monash University and Monash Heart, Monash Health Clayton Victoria Australia; ^6^ Department of Internal Medicine and Cardiovascular Center Seoul National University Hospital Seoul South Korea; ^7^ Department of Medicine and Radiology University of British Columbia Vancouver British Columbia Canada; ^8^ Centro Cardiologico Monzino, IRCCS, Milano ‐ Dipartimento di Scienze Cliniche e di Comunità Università degli Studi Milan Italy; ^9^ Department of Advanced Biomedical Sciences University Federico II Naples Italy; ^10^ Clinical Research Institute for Clinical Pharmacology and Therapeutics Showa University Tokyo Japan; ^11^ HeartFlow Inc Redwood City USA; ^12^ Department of Cardiology University Hospital of Lausanne Lausanne Switzerland

## Abstract

**Introduction:**

Fractional flow reserve (FFR) measured after percutaneous coronary intervention (PCI) has been identified as a surrogate marker for vessel related adverse events. FFR can be derived from standard coronary computed tomography angiography (CTA). Moreover, the FFR derived from coronary CTA (FFR_CT_) Planner is a tool that simulates PCI providing modeled FFR_CT_ values after stenosis opening.

**Aim:**

To validate the accuracy of the FFR_CT_ Planner in predicting FFR after PCI with invasive FFR as a reference standard.

**Methods:**

Prospective, international and multicenter study of patients with chronic coronary syndromes undergoing PCI. Patients will undergo coronary CTA with FFR_CT_ prior to PCI. Combined morphological and functional evaluations with motorized FFR hyperemic pullbacks, and optical coherence tomography (OCT) will be performed before and after PCI. The FFR_CT_ Planner will be applied by an independent core laboratory blinded to invasive data, replicating the invasive procedure. The primary objective is to assess the agreement between the predicted FFR_CT_ post‐PCI derived from the Planner and invasive FFR. A total of 127 patients will be included in the study.

**Results:**

Patient enrollment started in February 2019. Until December 2020, 100 patients have been included. Mean age was 64.1 ± 9.03, 76% were males and 24% diabetics. The target vessels for PCI were LAD 83%, LCX 6%, and RCA 11%. The final results are expected in 2021.

**Conclusion:**

This study will determine the accuracy and precision of the FFR_CT_ Planner to predict post‐PCI FFR in patients with chronic coronary syndromes undergoing percutaneous revascularization.

## 1 INTRODUCTION

Fractional flow reserve (FFR), an invasive measurement of epicardial conductance during hyperemia, quantifies the amount of flow reduction due to epicardial narrowing and correates with myocardial ischemia.^1^ Coronary flow can be restored by revascularization. Percutaneous coronary intervention (PCI) is an effective method to improve myocardial perfusion and relieve patients from angina.^2^, ^3^, ^4^ The International Study of Comparative Health Effectiveness With Medical and Invasive Approaches (ISCHEMIA) study confirmed the clinical benefit of revascularization in terms of relieve from angina compared with a conservative strategy; nonetheless, the invasive strategy showed no benefit concerning the occurrence of adverse cardiovascular events in stable patients at 3 years follow‐up.^4^

After a successful PCI, approximately one fourth of patients remain with impaired coronary flow.^5^ The degree of functional revascularization can be assessed invasively by measuring FFR immediately after stent implantation. Complete functional revascularization (i.e. high post‐PCI FFR) has been associated with improved clinical outcomes after PCI ^6^ whereas low post‐PCI FFR has been identified as an independent predictor of vessel‐related adverse events.^7^ Therefore, a tool that predicts improvement in epicardial conductance would be of benefit for clinical decision making about revascularization and procedural planning.

Coronary computed tomography angiography (CTA) allows for the evaluation of coronary artery disease (CAD) in the non‐invasive setting.^8^ Coronary geometries derived from CTA can be utilized to perform blood flow simulation and estimate FFR. FFR derived from CT (FFR_CT_) has been shown to be accurate compared with invasive FFR.^9^ The FFR_CT_ Planner (HeartFlow, Inc., Redwood City, CA) is a novel tool able to recompute FFR_CT_ values after opening coronary stenoses. The Planner leverages the results of multiple simulations and reduced order modeling to instantly calculate a FFR_CT_ value in the desired lumen configuration. This provides the benefit of anticipating the effect of PCI influencing treatment planning prior to the catheterization laboratory.^10^ The hypothesis of the present study is that the FFR_CT_ Planner would be accurate and precise in predicting the results of PCI in terms of coronary physiology. Thus, the present study aims to validate the performance of the FFR_CT_ Planner to predict FFR post PCI with invasive FFR as a reference standard.

## 2 METHODS

### 2.1 Study design

The PRECISE PCI PLAN (P3) study is an investigator‐initiated, prospective, multi‐center study evaluating the diagnostic accuracy of FFR_CT_ Planner. Patients with chronic coronary syndromes with invasive FFR≤0.80 in at least one vessel and guideline‐directed indication for PCI will be eligible for inclusion. Table 1 shows inclusion and exclusion criteria. Prior to PCI, all patients will undergo coronary CTA with calculation of FFR_CT_. Invasive FFR will be followed by a motorized hyperaemic pullback evaluation; this will be performed before and repeated after PCI using a dedicated acquisition protocol.^11^ In addition, optical coherence tomography (OCT) will be used to guide PCI and optimize stent implantation. PCI optimization either based on FFR pullbacks and/or OCT will be allowed at the discretion of the operators. Each participating center will undergo peer‐to‐peer review of coronary CTA, angiography, OCT, FFR and motorized FFR standards before initiation of study enrollment. The study protocol has been approved at each participating center by the local Ethics Committee. All study subjects provide written informed consent prior to undergoing any study‐specific procedures. This study is registered as NCT03782688.

**TABLE 1:** Inclusion and exclusion criteria

Inclusion criteria	Exclusion criteria
Age ≥ 18 years Willing and able to provide written informed consent Having Coronary CTA with sufficient quality to allow for FFR_CT_ processing Having evidence of myocardial ischemia with an invasive FFR ≤0.80 and amenable to PCI	Severely calcified lesion/vessel Bifurcation lesions Ostial lesions Left main disease Severe vessel tortuosity^a^ Chronic obstructive pulmonary disease Contraindication to adenosine NYHA class III or IV, or last known left ventricular ejection fraction <30% Uncontrolled or recurrent ventricular tachycardia Atrial fibrillation, flutter or arrhythmia History of recent stroke (≤90 days) History of acute coronary syndrome (≤90 days) Prior myocardial infarction History of ischemic stroke (>90 days) with modified RANKIN score ≥ 2 History of any hemorrhagic stroke Previous revascularization (PCI or CABG) Active liver disease or hepatic dysfunction, defined as AST or ALT >3 times the ULN Severe renal dysfunction, defined as an eGFR <30 ml/min/1.73 m^2^ BMI > 35 kg/m^2^ Nitrate intolerance Contra‐indication to heart rate lowering drugs Insufficient coronary CTA image quality assessed by an independent committee.

^a^ Tortuosity was defined as one or more bends of 90° or more, or three or more bends of 45° to 90° proximal of the diseased segment.

The study leadership is composed by a principal investigator, a co‐principal investigator, a chairman and steering committee. Clinical events will be adjudicated by an independent clinical events committee. Imaging (i.e. coronary CTA, invasive coronary angiography and OCT) and physiology data (i.e. hyperaemic pressure tracings) will be analyzed by an independent core‐laboratory. In addition, the quality of the coronary CTA images will be assessed by an independent CT quality committee. Details of each of these committees and their members are shown in the Supplemental Appendix.

### 2.2 Primary and secondary endpoint

The primary endpoint is to assess the agreement between FFR_CT_ Planner and invasively measured FFR post‐PCI. The secondary endpoints include: (a) Comparison between non‐invasive and invasive FFR pullbacks pre‐ and post‐PCI. (b) Comparison of changes in lesion gradient from pre‐ to post‐PCI between FFR_CT_ and invasive FFR. (c) Comparison of luminal dimensions derived from FFR_CT_ Planner with OCT post‐PCI . (d) To assess the presence and severity of residual angina assessed by the Seattle angina questionnaire (SAQ‐7) stratified by post‐PCI FFR_CT_ at 6 to 12 months follow‐up.

### 2.3 Study logistics

Coronary CTA and FFR_CT_ will be performed as part of standard of care. Once eligibility has been confirmed, patients will be invited to participate in the study. PCI will be performed following a dedicated protocol including combined invasive FFR and OCT evaluation for procedural guidance and stent optimization. Morphological and functional data will be centrally collected by the core laboratory (CoreAalst BV, Aalst, Belgium) for analysis. The FFR_CT_ diagnostic model with the position of the stent, derived from the invasive coronary angiography, will be sent to the FFR_CT_ core laboratory (HeartFlow Inc, Redwood city, California, US) to apply the FFR_CT_ Planner blinded to the invasive data. All data will be centrally processed, analyzed and co‐registered by the central core laboratory (Supplemental figure).

### 2.4 Coronary CTA analysis

The coronary CTA will be performed using contemporary single‐ and dual‐source CT scanners with a minimum of 128 detector rows and gantry rotation times <330 milliseconds. Laboratories will follow local CT acquisition protocols being in accordance with quality standards defined by the Society of Cardiovascular Computed Tomography.^12^ Oral or intravenous beta‐blockers will be administered to achieve heart rate ≤ 65 bpm. Before the scan, patients will receive nitrates to ensure coronary vasodilatation. The coronary CTA images will be transferred to an independent core laboratory for analysis. Non‐invasive quantitative coronary analysis (NI‐QCA) will be performed using the luminal dimensions from the FFR_CT_ model.^13^ Lesion length will be defined as the length between normal ( without plaque) proximal and distal reference segments. Minimal lumen area, proximal and distal reference lumen areas, and area stenosis will be assessed. The quality of coronary CTA will be adjudicated using a four‐points Likert score at the vessel level.

### 2.5 FFR derived from CT

**FIGURE 1:**
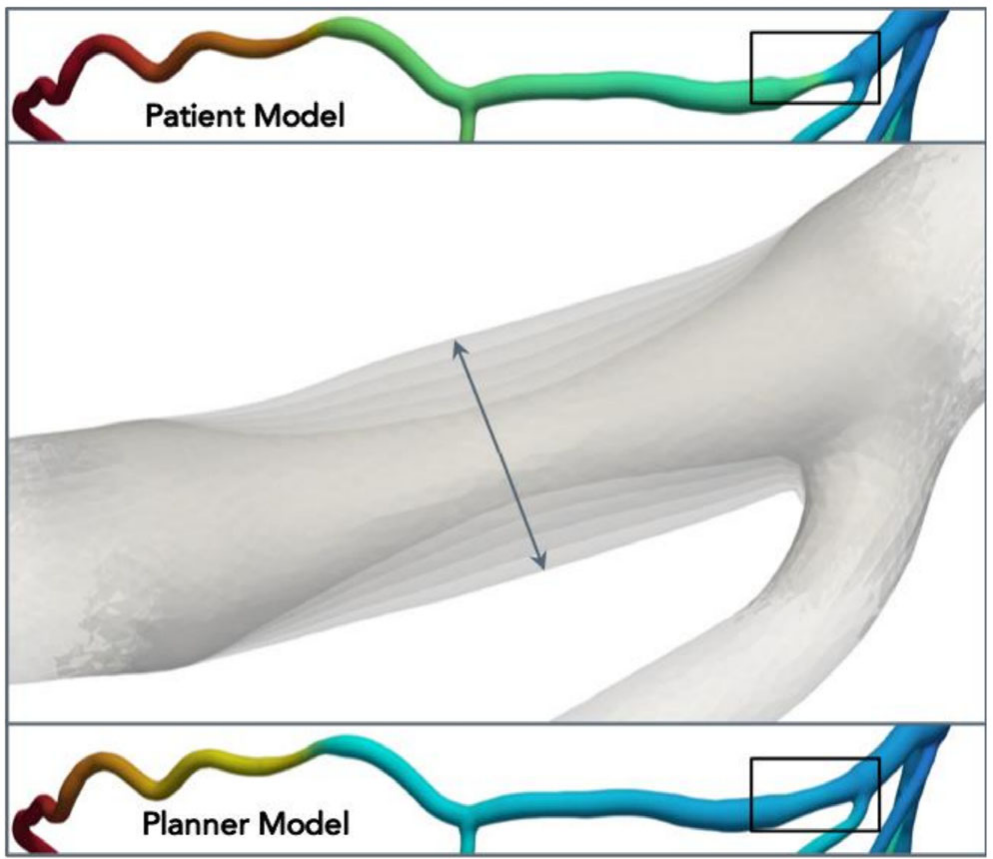
Example of lumen modeling using the FFR_CT_ planner. On the top panel, patient‐specific model showing a severe stenosis in the proximal segment of the vessel (black square) with the corresponding colored‐coded changes in FFR_CT_. The mid panel shows the luminal remodeling process. In the bottom panel, the remodeled geometry with the corresponding changes in FFR_CT_ values. FFR_CT_ Fractional flow reserve derived from CT. FFR Fractional flow reserve

Coronary CTA datasets will be processed for FFR_CT_ using a validated method (HeartFlow, Inc, Redwood City, California, USA). Briefly, models will be constructed from automated algorithms and trained analysts. Blood flow simulations will be performed on patient‐specific coronary geometries to compute FFR_CT_ values.^14^ The FFR_CT_ Planner will be applied blinded to the invasive functional data to remodel the lumen and provide a FFR_CT_ value after stenosis removal (Figure 1). For the primary endpoint, the FFR_CT_ value matching the invasive pressure wire sensor position will be used. FFR_CT_ values will be extracted at every 0.1 mm to create FFR_CT_ pullback curves for co‐registration with invasive motorized FFR pullbacks.

### 2.6 Coronary angiography

Invasive coronary angiography will be performed following a dedicated protocol. Briefly, intracoronary nitroglycerin injection (100‐200 μg) will be administered before angiography. At least two projections separated by at least 30° will be obtained before and after PCI. Stent position before implantation will be recorded in two projections facilitating co‐registration with the FFR_CT_ model. Coronary angiography will be analyzed with 3D‐QCA software (CAAS 8.2 Software, Pie Medical Imaging, Maastricht, The Netherlands). In case of lesions involving coronary bifurcations dedicated QCA bifurcation software packages will be used.^15^ Minimum lumen area, percent area stenosis, reference vessel areas and lesion length will be reported. QCA analysis will be performed by an independent core laboratory.

### 2.7 Fractional flow reserve

A sensor‐tipped 0.014‐inch pressure guidewire (Pressure wire X, Abbott Vascular, Chicago, IL, USA) will be introduced into the target vessel. The sensor will be located in the distal segment of the coronary artery with a diameter ≥ 2 mm by visual estimation within 13 cm from the coronary ostium. The sensor should be located at least 20 mm beyond the most distal stenosis by visual estimation. The pressure wire position will be recorded using a contrast injection for co‐registration purposes. Hyperemia will be obtained with intravenous adenosine administrated at a rate of 140 μg/kg/min preferentially via a central vein for at least 2 min to obtain a steady hyperemic state. Invasive FFR measurements will be performed pre‐ and post‐PCI at the same anatomical location. In addition, FFR pullbacks will be performed using a motorized device (R 100, Volcano, San Diego, Ca, USA) at a speed of 1 mm per second. The standardization of the pressure and vessel length relationship will allow for co‐registration of the pressure along the coronary vessel. Pressure tracings and pullback curve quality will be assessed by an independent core laboratory. Functional gain will be defined as the FFR post‐PCI minus the FFRpre‐PCI. Lesion gradients will be defined as the FFR values at the proximal edge of the stent minus the FFR at the distal edge of the stent. To quantify the pattern of coronary artery disease (e.g. focal or diffuse), the pressure pullback gradient (PPG) will be calculated. Analysis of invasive functional data will be performed using Coroflow software (Coroventis research, Uppsala, Sweden).

### 2.8 Optical coherence tomography

**FIGURE 2:**
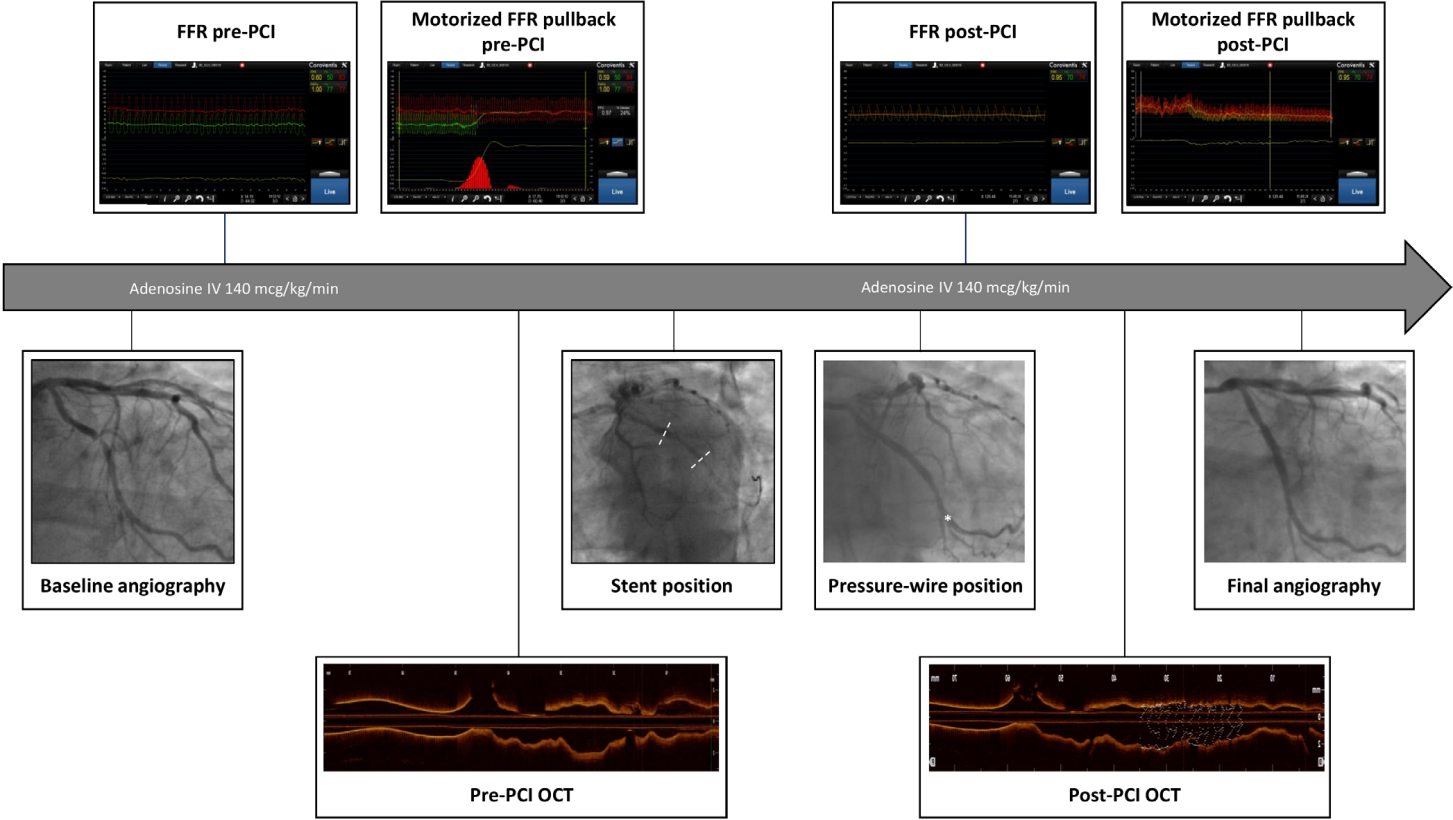
Invasive procedure steps. After acquisition of the baseline angiography, pre‐PCI FFR is measured followed by a motorized pullback evaluation. The wire position for the distal FFR measurement is recorded with a contrast injection for off‐line co‐registration with the FFR_CT_ model. Subsequently, OCT is performed to assess lesion characteristics, define stent size and PCI strategy. The position of the stent, before deployment, is recorded with a contrast injection to co‐localize the stent position in the FFR_CT_ model for application of the Planner (dashed white line). Following PCI, OCT is performed to assess final stent expansion and apposition. FFR and motorized pullback evaluation are then repeated. The position of the pressure sensor is recorded with a contrast injection (white star). The procedure is completed with final coronary angiography of the target vessel. FFR_CT_, fractional flow reserve derived from CT. FFR Fractional flow reserve; OCT, optical coherence tomography

Optical coherence tomography (Abbott Vascular, St. Paul, Minnesota) will be acquired pre‐ and post‐PCI. OCT images will be used online to guide PCI and optimize stent implantation at operator discretion. Lesion length will be based on normal‐to‐normal landing zones in the pre‐PCI OCT. Minimum lumen area, reference lumen areas and lesion length will be analyzed.^16^ Post PCI, minimum stent area (MSA) and stent expansion will be reported. Stent expansion will be defined as MSA in both the proximal and distal halves of the stent relative to the closest reference segment. OCT images will be analyzed by an independent core laboratory.. Figure 2 shows angiographic, FFR and OCT acquisition protocol.

### 2.9 Co‐registration

**FIGURE 3:**
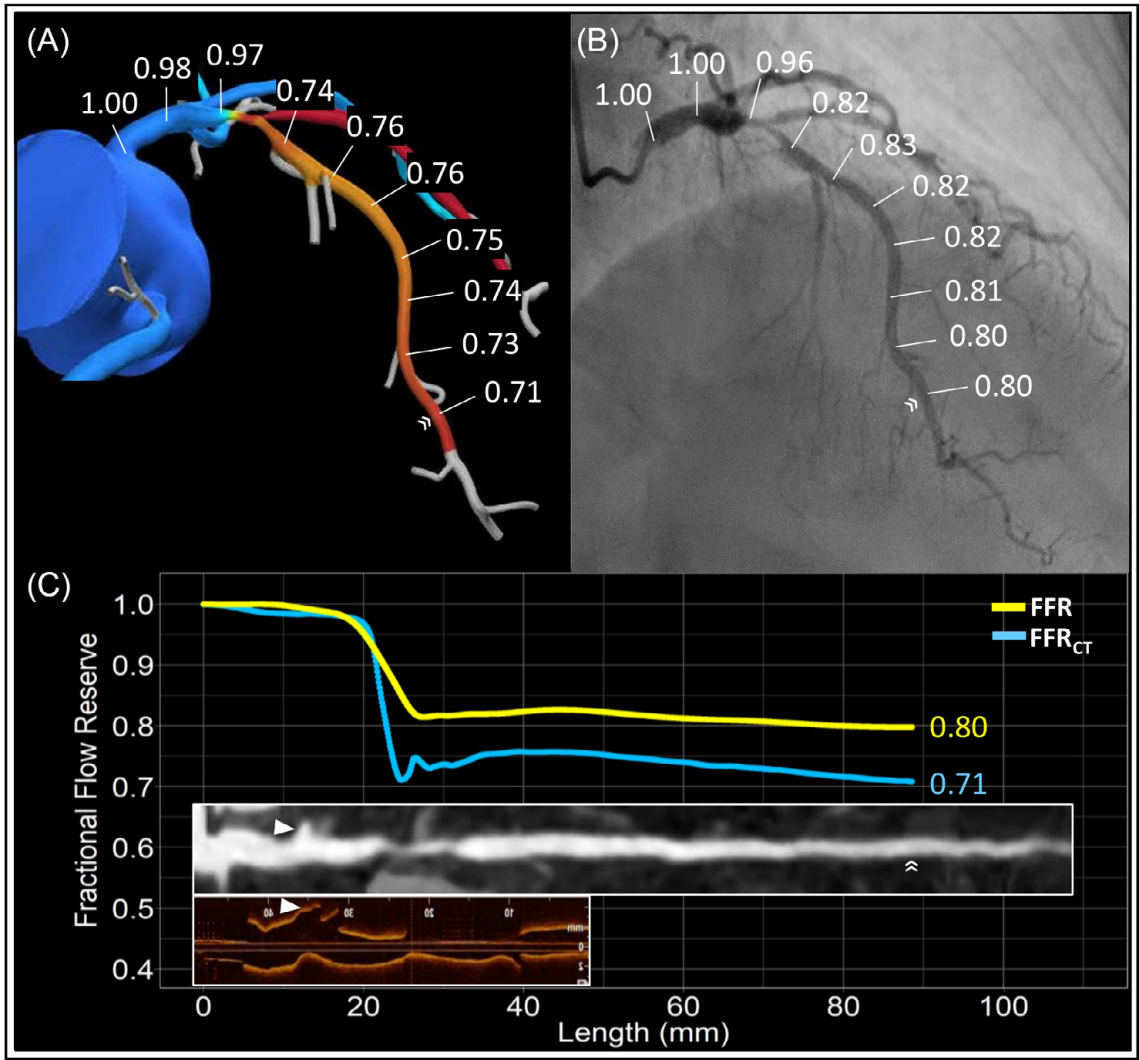
Co‐registration of FFR_CT_, FFR, Coronary CTA and OCT. Panels A and B show a left anterior descending artery with the corresponding FFR_CT_ and FFR. The FFR_CT_ and FFR values along the vessel are used to generate pullback curves shown in panel C in blue and yellow, respectively. The white double arrows point the location of the pressure wire sensor used to co‐register invasive and non‐invasive functional data. On the bottom of panel C, a CT straight multiplanar reconstruction and OCT longitudinal view are co‐registered with the physiologic data. The white arrow heads show the position of side branches used to co‐register OCT and coronary CTA. Coronary CTA coronary computed tomography angiography. FFR_CT_, fractional flow reserve derived from CT. FFR Fractional flow reserve; OCT, optical coherence tomography

**FIGURE 4:**
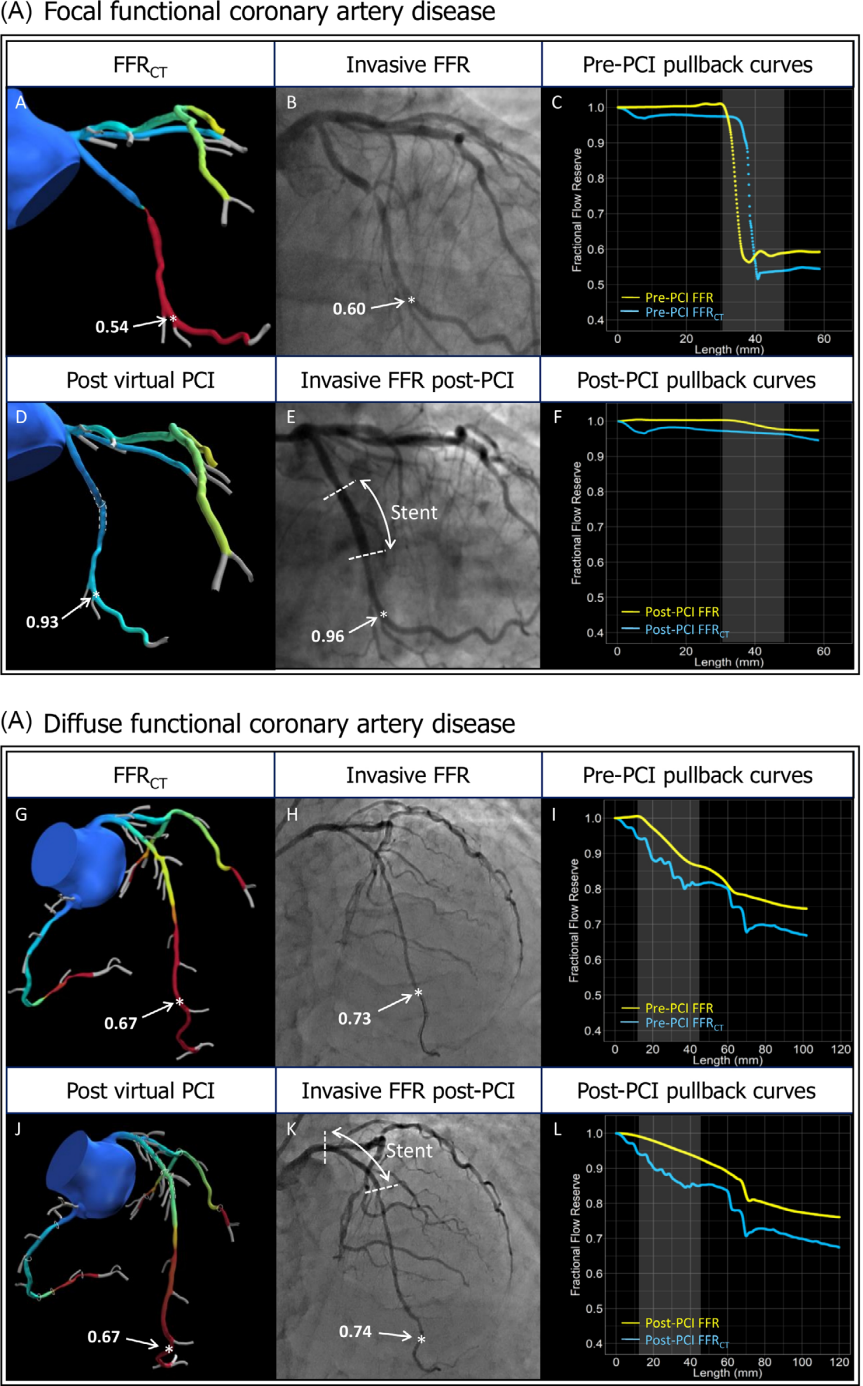
Case example of the application of FFR_CT_Planner incases with focal and diffuse functional coronary artery disease. (A) Focal functional coronary artery disease. Panel A shows the FFR_CT_ model showing a focal, hemodynamic significant lesion in the Circumflex coronary artery. Panel B shows invasive angiography confirming an angiographic focal lesion. The position of the pressure wire sensor is denoted by a white star. Panel C shows the FFR_CT_ and invasive FFR pullback curves. Panel D shows the remodeled geometry (white dashed lines) and presents the results of the blinded luminal remodeling using the FFR_CT_ Planner. Panel E shows the location of distal invasive FFR assessment post‐PCI (white star) matched with the FFR_CT_ model. The FFR_CT_ Planner predicted a FFR_CT_ value of 0.93 at the same position (white star) where the invasive FFR post‐PCI recorded 0.96. Panel F shows the corresponding post‐PCI pullback curves derived from FFR_CT_ and invasive FFR (blue and yellow lines, respectively). (B) Diffuse functional coronary artery disease. Panel G shows a patient‐specific FFR_CT_ model with diffuse pressure loss along the LAD and distal FFR_CT_ value of 0.67. Panel H shows invasive coronary angiography with distal invasive FFR value of 0.73 (white stars). Panel I shows the FFR_CT_ and FFR pullback curves pre‐PCI. Panel J shows the remodeled segment in the FFR_CT_ model (white dashed lines) predicting a FFR of 0.67. Panel K shows the location of the invasive FFR measurement of 0.74 (white star). Panel L shows the post‐PCI pullback curves derived from FFR_CT_ and invasive FFR (blue and yellow lines, respectively). FFR_CT_ Fractional flow reserve derived from CT. FFR, fractional flow reserve; LAD, left anterior descending artery; PCI, percutaneous coronary intervention

Coronary imaging data from coronary CTA, invasive angiography and OCT will be matched using fiduciary points. In addition, physiological data from invasive FFR and FFR_CT_ pullbacks will be co‐localized. Furthermore, morphological (i.e., coronary CTA, angiography and OCT) and physiologic (FFR_CT_ and invasive FFR) will be co‐registered. For co‐registration, vessels will be divided in three segments (i.e., proximal, lesion and distal). The proximal segment will be defined as from the ostium to the proximal lesion edge. The lesion will be defined as the stented segment, and the distal segment will be defined from the distal stent edge to the position of the pressure sensor. Co‐registration of invasive and non‐invasive FFR pullbacks will be performed as previously described.^17^ Co‐registration between coronary CTA and OCT will be performed using a proprietary automated algorithm based on side branches location. . Figure 3 shows an example of the co‐registration process. Case examples are shown in Figure 4.

### 2.10 Clinical outcomes

Clinical follow‐up will be performed in‐hospital, at 6 ± 1 month and 1 year after the procedure. Peri‐procedural myocardial infarction will be defined according to the Fourth Universal definition.^18^ The rate of TVF and its components (cardiac death, target vessel myocardial infarction, ischemia driven target vessel revascularization) and stent thrombosis will be assessed at 6 months, 1 year and yearly until 5 years follow‐up. Seattle angina questionnaire‐7 items (SAQ‐7) will be administrated at 6 to 12 months after PCI. Adverse events will be adjudicated by an independent clinical events committee.

### 2.11 Statistical analysis and sample size calculation

The P3 study will assess the agreement between the FFR_CT_ Planner and invasively measured FFR after stent implantation. The agreement will be assessed using the Bland–Altman method. Mean difference or bias will be considered a metric of accuracy, and standard deviation as a metric of precision. Based on prior data, the mean difference between FFR_CT_ Planner and invasive FFR post PCI is assumed to be 0.04 FFR units with a standard deviation of 0.07.^19^ With these assumptions and confidence levels of 0.95 and power of 80% a sample size of 123 vessels would be required. Assuming an attrition rate of 2.5%, 127 vessels will be included. The P3 study is not statistically powered to assess its secondary endpoints.

### 2.12 Planned sub‐studies

In addition to the primary and secondary objectives, several sub‐studies are planned. Briefly, we will (1) assess the accuracy of the FFR_CT_ Planner stratified by coronary CTA image quality; (2) assess the relationship between the pre‐PCI pattern of functional coronary artery disease (e.g. focal or diffuse) quantified by the PPG and post‐PCI FFR using both invasive and non‐invasive FFR pullbacks; (3) assess the relationship between luminal dimensions derived from coronary CTA and OCT, and (4) we planned to assess the relationship between plaque type and trans‐lesional pressure gradients using coronary CTA and iOCT.

## 3 RESULTS

Patient enrollment started in February 2019, until December 2020, 100 patients have been included. Mean age was 64.1 ± 9.03, 76% were males and 24% diabetics. The target vessels for PCI were LAD 83%, LCX 6% and RCA 11%. Table 2 shows the baseline characteristics of the population included. Recruitment is ongoing and it is anticipated that the primary results will be presented in Summer 2021.

**TABLE 2:** Preliminary baseline characteristics

	N = 100
Age, years, median [IQR]	64.1 ± 9.03
Gender male, n (%)	76 (76.0)
Coronary risk factors	
Diabetes, n (%)	24 (24.0)
Dyslipidemia, n (%)	79 (79.0)
Hypertension, n (%)	54 (54.0)
Current smoking, n (%)	21 (21.0)
Prior PCI, n (%)	6 (6.0)
Peripheral vascular diease, n (%)	3 (3.0)
Prior stroke, n (%)	3 (3.0)
Clinical Presentation, n (%)	
Silent ischemia	24 (24.0)
Stable angina CCS I	35 (35.0)
Stable angina CCS II	28 (28.0)
Stable angina CCS III	8 (8.0)
Stable angina CCS IV	1 (1.0)
Unstable angina	4 (4.0)
Vessel, n (%)	
LAD	83 (83.0)
LCX	6 (6.0)
RCA	11 (11.0)

## 4 DISCUSSION

The present study will assess the accuracy and precision of the FFR_CT_ Planner to predict the degree of functional revascularization with invasive FFR post PCI as reference. The availability of tools that predict the results of PCI in terms of coronary physiology is expected to impact the field of interventional cardiology improving patient selection and PCI strategies. Furthermore, the FFR_CT_ Planner is based on coronary CTA, a modality which is increasingly used to evaluate patients with suspected coronary artery disease in the non‐invasive setting. Therefore, this novel tool may increase the use of coronary CTA for planning percutaneous revascularization procedures.^20^

The accuracy of FFR_CT_ has been established with invasive pre‐PCI FFR as the reference standard ^9^. The P3 Study expands the investigation of the FFR_CT_ technology by assessing the accuracy of the FFR_CT_ Planner compared to post‐PCI FFR. Until now, three studies have assessed the performance of the FFR_CT_ Planner. Kim et al. in 44 patients reported mean difference between the FFR_CT_ Planner and FFR post‐intervention of 0.024 (95% limit of agreement: −0.08 to 0.13).^21^ More recently, Bom et al. in 56 patients observed a mean difference in post‐PCI FFR between FFR_CT_ Planner and invasive FFR of 0.040 (95% limit of agreement: −0.10 to 0.18).^19^ Furthermore, in patients with angiographic serial lesions the FFR_CT_ Planner showed to accurately predict the true FFR translesional gradients.^22^ The abovementioned studies have suggested that the FFR_CT_ Planner is accurate to predict post‐PCI FFR; nonetheless, these were limited because of their single‐center design, post‐hoc analyses of other studies and the lack of statistical power to assess the performance of this tool.

The combination of coronary CTA with its ability to assess atherosclerotic plaque extent, and FFR_CT_ allowing to assess pressure losses along the coronary vessel provides a unique opportunity to evaluate the anatomical and functional CAD patterns. Two predominant phenotypes of coronary artery (i.e. focal or diffuse) have been described.^23^, ^24^ In cases of focal functional CAD with lesion‐specific ischemia, PCI often results in complete functional revascularization. In contrary, in cases of diffuse CAD, no focal pressure gradients are present despite the presence of one or more angiographic stenoses. In the latter, PCI results are frequently sub‐optimal in terms of post‐PCI FFR whereas in the former PCI restore epicardial conductance. FFR_CTP_ lanner will facilitate the integration of a comprenhensive functional assessement for PCI planning.

At variance with diagnostic FFR_CT_, the FFR_CT_ Planner is designed to be used in patients with significant CAD. The FFR_CT_ Planner could be used at two phases of the evaluation of patients with CAD. First, to determine the suitability for percutaneous revascularization. Patients with diffuse disease, for example, showing negligible functional improvement with PCI could be informed of the anticipated results, the likelihood of angina relief and other more suited therapeutics options. Second, in the catheterization laboratory, the FFR_CT_ Planner may help in assessing the location of pressure losses, length of coronary segments to be treated to achieve optimal post‐PCI FFR values and tailor your PCI strategy in cases of serial lesions. By this, the FFR_CT_ Planner has the potential to increase the degree of complete functional revascularization.

### 4.1 Limitations

The present study has several limitations. First, the evaluation of the performance of the FFR_CT_ Planner is based on its accuracy compared to invasively measured FFR. Post‐PCI FFR is a surrogate of clinical outcomes after percutaneous revascularization. The study, however, is not powered to assess clinical outcomes. Second, the information of the FFR_CT_ planner will not be used to guide PCI; thus, the clinical impact of the prospective application of this technology remains to be determined. Third, the sample size is relatively small; nevertheless, powered to assess the accuracy and precision of the FFR_CT_ planner. Fourth, stent optimization will be guided by FFR pullbacks and OCT which may not represent routine clinical practice.

## 5 CONCLUSION

This prospective and multicenter study will determine the accuracy and precision of the FFR_CT_ Planner to predict post‐PCI FFR. Prediction of post‐PCI FFR may improve patient selection for percutaneous revascularization, anticipate the clinical benefit of the intervention and refine the revascularization strategy. A larger clinical trial will be required to assess the impact of the FFR_CT_ Planner guided strategy on clinical outcomes.

## CONFLICT OF INTEREST

JS report research grants provided by Cardiopath PhD program. BDB reports receiving consultancy fees from Boston Scientific, and Abbott and receiving research grants from Coroventis Research, Pie Medical Imaging, Cathworks, Boston Scientific, Siemens, HeartFlow Inc. and Abbott Vascular. CC reports receiving research grants from Biosensor, Coroventis Research, Medis Medical Imaging, Pie Medical Imaging, Cathworks, Boston Scientific, Siemens, HeartFlow Inc. and Abbott Vascular; and consultancy fees from Heart Flow Inc, Opsens, Abbott Vascular and Philips Volcano. WH reports that Cardiac Research Institute Aalst receives consultancy fees on his behalf from Boston Scientific, St Jude Medical, Microport, Medtronic, Biotronik, Astra Zeneca. AU, CR and CT are employees of HeartFlow Inc. JL is a consultant and holds stock options in Circle CVI and Heartflow. Research grant from GE and modest speaker fees for GE and Philips. BLN and JMJ have received unrestricted institutional research grants from Siemens and HeartFlow. HO reports receiving research grants from Abbott Vascular; and speaker fees for Heartflow and Abbott Vascular. BK has received consulting fees from Canon Medical, Abbott and Medtronic. AI has received consulting fees from Canon, Artrya Medical and Boston Scientific. BKK report institutional research grants provided by HeartFlow, Inc. DA report research grants from GE healthcare and Bracco. EB received speaker's fees from Boston Scientific, Abbott Vascular and GE. MM received advisory board and/or lecture fees from AstraZeneca, Bayer, Boehringer‐Ingelheim, Bristol Myers‐Squibb, Boston Scientific, and Novo Nordics, and research grants from Bayer and Volcano. The authors have nothing to disclose.

## Supporting information


**Appendix S1.** Supporting Information.

**Supplemental figure 1 Study Logistics** After the eligibility of patients with suspected coronary artery disease with FFR_CT_ ≤ 0.80 is assessed, combined FFR and OCT guided PCI will be performed. All data will be collected by core laboratory for analysis. The FFR_CT_ diagnostic model with the stent position will be sent to HeartFlow Inc (Redwood city, California, US) and the FFR_CT_ Planner blinded to the invasive data will be sent back to core laboratory. Finally, the agreement of post‐PCI FFR and FFR_CT_ planner will be investigated. FFR_CT_ Fractional flow reserve derived from CT. FFR Fractional flow reserve. OCT Optical coherence tomography. PCI percutaneous coronary intervention.

## Data Availability

The data that support the findings of this study are available from the corresponding author upon reasonable request.
