# Supplementary material for: Rationale and design of the precise percutaneous coronary intervention plan (P3) study: Prospective evaluation of a virtual computed tomography‐based percutaneous intervention planner
Source: Clin Cardiol. 2021 Mar 3;44(4):446–54. doi: 10.1002/clc.23551 (PMC8027584; doi:10.1002/clc.23551)
Supplement: Supplementary file 1 — Appendix S1. Supporting Information. [file CLC-44-446-s001.docx]

The participating centres and principal investigators:

1. Jeroen Sonck, Cardiovascular Center Aalst, OLV Clinic
2. Bjarne Norgaard, Aarhus University, Aarhus, Denmark.
3. Bon-Kwon Koo, Seoul National University, Korea.
4. Brian Ko, Monash Health, Melbourne, Australia.
5. Hiromase Otake, Kobe, Japan

Steering committee:

1. Prof. Bernard de Bruyne (Chairman)
2. Jeroen Sonck (Principal Investigator)
3. Carlos Collet Bortone (Co-Principal Investigator)
4. Bjarne Norgaard (Principal Investigator)
5. Bon-Kwon Koo (Principal Investigator)
6. Brian Ko (Principal Investigator)
7. Hiromase Otake (Principal Investigator)
8. Jonathon Leipsic
9. Emanuele Barbato
10. Joseph Bartunek
11. Charles Taylor
12. Campbell Rogers
13. Daniele Andreini

Clinical Event Committee (CEC)

1. Ward Heggermont
2. Dan Schelfaut
3. Jan Leeman

Coronary CTA image quality committee

1. Daniele Andreini
2. Martin Penicka
3. Lieven Van Hoe

Core Laboratory

1. Takuya Mizukami
2. Sakura Nagumo
3. Niya Mileva
4. Daniel Munhoz
