# Supplementary figures and images for: Rationale and design of the precise percutaneous coronary intervention plan (P3) study: Prospective evaluation of a virtual computed tomography‐based percutaneous intervention planner
Source: Clin Cardiol. 2021 Mar 3;44(4):446–54. doi: 10.1002/clc.23551 (PMC8027584; doi:10.1002/clc.23551)

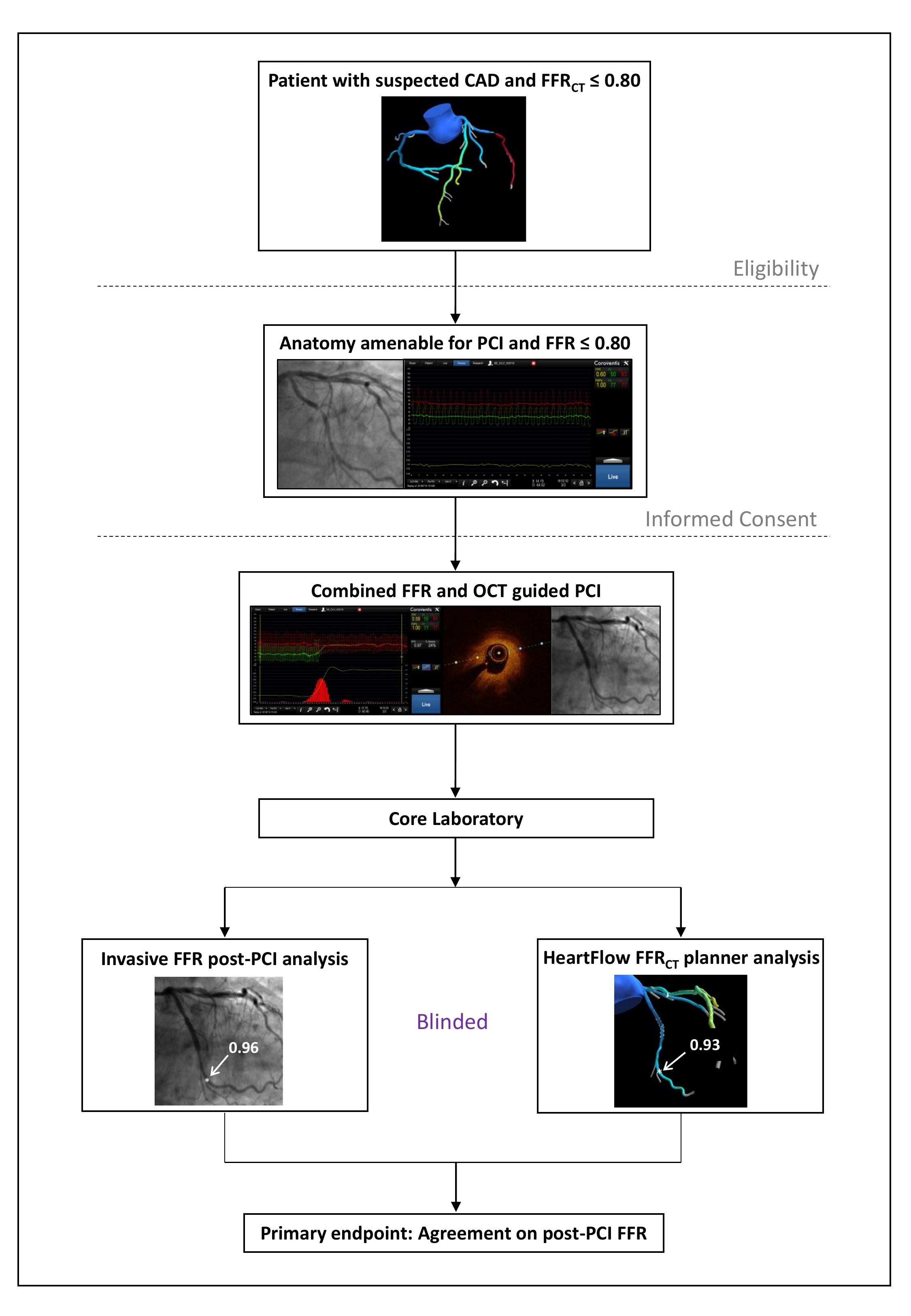

Supplement: Supplementary file 2 — Supplemental figure 1 Study Logistics After the eligibility of patients with suspected coronary artery disease with FFRCT ≤ 0.80 is assessed, combined FFR and OCT guided PCI will be performed. All data will be collected by core laboratory for analysis. The FFRCT diagnostic model with the stent position will be sent to HeartFlow Inc (Redwood city, California, US) and the FFRCT Planner blinded to the invasive data will be sent back to core laboratory. Finally, the agreement of post‐PCI FFR and FFRCT planner will be investigated. FFRCT Fractional flow reserve derived from CT. FFR Fractional flow reserve. OCT Optical coherence tomography. PCI percutaneous coronary intervention. [file CLC-44-446-s002.jpg]
